# Knowledge of Internal Quality Control for Laboratory Tests among Laboratory Personnel Working in Department of Biochemistry in a Tertiary Care Center: A Descriptive Cross-sectional Study

**DOI:** 10.31729/jnma.8040

**Published:** 2023-02-28

**Authors:** Bijaya Mishra, Binod Kumar Lal Das, Seraj Ahmed Khan, Basanta Gelal, Apeksha Niraula, Rajendra Kumar Chaudhari, Madhab Lamsal

**Affiliations:** 1 Department of Biochemistry, B.P. Koirala Institute of Health Sciences, Ghopa, Dharan, Nepal; 2 Department of Biochemistry, Tribhuwan University, Institute of Medicine, Maharajgunj, Kathmandu, Nepal

**Keywords:** *biochemistry*, *knowledge*, *laboratory personnel*, *quality control*

## Abstract

**Introduction:**

Clinical laboratory holds a central position in patient care, thus, ensuring accurate laboratory test results is a necessity. Internal quality control ensures day-to-day laboratory consistency. However, unless practised, laboratory quality systems cannot be achieved. This depends on the efforts and commitment of laboratory personnel for its implementation. Hence, the aim of this study was to find out the knowledge of internal quality control for laboratory tests among laboratory personnel working in the Department of Biochemistry in a tertiary care centre.

**Methods:**

This was a descriptive cross-sectional study conducted from 1 July 2022 to 30 August 2022 after receiving ethical approval from Institutional Review Committee (Reference number: 2341/022). Semi-structured questionnaire was used to assess knowledge on internal quality control. Three nonrespondents were excluded. The operational definition of the knowledge domain was set before finalizing the questionnaire. The convenience sampling method was used. Point estimate and 95% Confidence Interval were calculated.

**Results:**

Among 20 laboratory personnel, 5 (25%) (6.02-43.98, 95% Confidence Interval) had adequate knowledge of internal quality control. The mean knowledge score was 12±2.44.

**Conclusions:**

The prevalence of adequate knowledge of internal quality control for laboratory tests among laboratory personnel working in the Department of Biochemistry was similar to the other study done in a similar setting.

## INTRODUCTION

Clinical laboratory plays a central role in providing information about the health of patients for appropriate prevention, diagnosis and management of diseases.^1^ For confidence in reports generated within the laboratory, quality control (QC) plays a pivotal role.

QC which includes internal and external QC describes a set of procedures used to check if laboratory results are reliable for the intended clinical use. Internal quality control (IQC) ensures day-to-day laboratory consistency.^2^ Currently, the majority of laboratory results are generated by automated analyzers, with the shift in laboratory personnel's duties from manual work to equipment maintenance, internal and external QC, instrument calibration and data management of generated results.^3^ Quality systems in the laboratory cannot be achieved if not practised properly, and this ultimately depends upon the knowledge, efforts and commitment of laboratory personnel for its implementation.^4^

The objective of this study was to find out the knowledge of internal quality control for laboratory tests among laboratory personnel working in the Department of Biochemistry in a tertiary care centre.

## METHODS

A descriptive cross-sectional study was conducted in the Department of Biochemistry, at B.P. Koirala Institute of Health Sciences (BPKIHS), Dharan, Nepal after obtaining ethical approval from the Institutional Review Committee (Reference number: 2341/022). The data were collected from 1 July 2022 to 30 August 2022. Personnel working in laboratories under the Department of Biochemistry were included. The faculty in-charge, the technical in charge and those not willing to participate were excluded from the study. Convenience sampling was used. The sample size was calculated by using the following formula:


$$\begin{array}{ll} \text{n} & ={\text{Z}}^{2}\times \frac{\text{p}\times \text{q}}{{\text{e}}^{\text{2}}}\\ & =1.96^{2}\times \frac{0.50\times 0.50}{0.1^{2}}\\ & =97 \end{array}$$

Where,

n = minimum required sample size
Z = 1.96 at 95% Confidence Interval (CI)
p = prevalence taken as 50% for maximum sample size calculation
q = 1-p
e = margin of error, 10%

Thus, the calculated sample size was 97. The abovecalculated sample size is adjusted for a finite population as


$$\begin{array}{ll} \text{n'} & =\frac{\text{n}}{1+\frac{\text{n}-1}{\text{N}}}\\ & =\frac{\text{97}}{1+\frac{\text{97}-1}{\text{23}}}\\ & =19 \end{array}$$

Where

n'= adjusted sample size for a finite population
N = 97, total number of laboratory personnel

Thus, the final sample size was 19. However, A total of 20 sample size was included.

A semi-structured questionnaire was used for data collection. The questionnaire comprised two segments including a socio-demographic profile and knowledge questions in regard to IQC. Sociodemographic variables sex, education level, position held in the laboratory and laboratory quality control training ware taken. The operational definition of knowledge on IQC was set prior to finalising the questionnaire, and the questionnaire was designed and finalized based on it. Questions on knowledge of IQC were based on the understanding of the purpose of IQC, the types of control materials, various control charts, how and when IQC should be performed and interpretations of the Levey-Jennings Chart using the Westgard rule. There were a total of 20 questions to assess the IQC knowledge domain. The scores were calculated by assigning 1 point to each correct answer and zero to incorrect/don't know/blank answers. A score of ≥10 was assigned as adequate knowledge, and ≤9 was assigned as inadequate knowledge.^5^

The participants were provided with information regarding the purpose of the study and what each one was expected to do to minimize bias. The questionnaire was collected back within 24 hours. The confidentiality of each laboratory personnel filling out the questionnaire was maintained throughout the study. Data were entered and analyzed using Microsoft Excel 2011. Point estimate and 95% CI were calculated.

## RESULTS

Among 20 laboratory personnel, only 5 (25%) (6.0243.98, 95% Confidence Interval) had adequate knowledge of internal quality control with the mean knowledge score in this group as 12±2.44 (Table 1).

**Table 1:** The sociodemographic profiles of laboratory personnel with adequate knowledge (n= 5).

Characteristics	Category	n (%)
Age (years)	44.4±3.21 (40-48)	5 (100)
Gender	Male	5 (100)
Education	Diploma	1 (20)
	Bachelor	2 (40)
	Master	2 (40)
Designation	Technician	2 (40)
	Technologist	3 (60)
Working experience (years)	19.6±4.61 (14-25)	5 (100)
Received training on laboratory quality management		2 (40)
Received training in laboratory IQC (years)	<1	-
	2-5	-
	5-10	-
	>10	1 (20)
	Never	4 (80)

## DISCUSSION

When we structure a questionnaire-based study, the main focus remains on a certain topic and are unique to a particular setting and are designed for a specific issue and topic.^4^ With the vital role that the laboratory holds in all health systems and in regard to the information about patient's health, having confidence in the reports generated within the laboratory becomes crucial.^1^ IQC ensures day-to-day laboratory consistency and that the results from the laboratory test are reliable, however, it is firstly a check of precision (i.e., reproducibility) but not necessarily accuracy.^5^ Well-developed and planned IQC program will continuously help ensures that the reports generated within the laboratory are accurate, reliable and reproducible.^6^ However, if not practised and adopted, the laboratory quality system fails. The practice of IQC majorly depends upon the laboratory personnel for its implementation which ultimately depends upon their knowledge, attitude and practice behaviour on IQC.^4^ According to Stepwise Laboratory Improvement Process Towards Accreditation (SLIPTA), performing IQC is one requirement that the laboratory should meet to achieve standards.^7^

Our study findings suggested that the majority of the laboratory personnel had inadequate knowledge in regard to internal quality control. Very few (25%) laboratory personnel had adequate knowledge of IQC. This finding was in accordance with the study done, which states that the performance of day-to-day IQC practice is based on the knowledge and expertise obtained through job experience rather than sound knowledge.^4^ A similar explanation has also been found in one of the studies.^8^ The finding from the study done, also reported that laboratory application of their knowledge was obtained mostly by on-job experience.^9^ A similar explanation could be implied in our study. Although not explored in our study, the inadequate content about laboratory quality management teaching in B.Sc.Mlt curriculum could be one of the reasons for inadequate knowledge.^4^

The mean age and working experience of the laboratory personnel in our study were higher than in other studies.^4,10^ Despite their age and higher working experience, they were inadequately trained on IQC, with the majority of them never attaining any quality control training after their education completion. This finding was similar to one of the studies, where 59% of laboratory personnel admitting that they were inadequately trained.^10^ This might be due to an inadequate practice/policy of the institute and the laboratory to provide training to the laboratory personnel on a regular basis. Thus, strengthening the laboratory's internal quality control system through proper and regular training and education of laboratory personnel seems a dire need in the particular context. We did not study the association of age and working experience on the average knowledge score, however, there are studies which show a positive association between these domains.^8^ A study found that more than 33% of laboratory personnel fail to practice IQC.^11^ In order to sustain improvements in the quality of laboratories, a regular survey on medical laboratories should be conducted which would question the accuracy and precision of laboratory analysis.^12^

Having regular IQC and total quality management training and meetings, conducting frequent and timely seminars/CMEs on the topic and motivating a reward system within a laboratory to allow more participation during such training and educational sessions, could be utilized as a strategy to enhance the knowledge amongst laboratory personnel. Numerous educational seminars, motivational sessions and improvement of IQC are needed to promote IQC use to reduce laboratory error in each step.^8^

The major factor for good IQC knowledge and practice is multifactorial. It involves education level, work experience, responsibility, and participation in various Accreditation programs and Laboratory Quality Management Systems (LQMS).^8^ In order to achieve day-to-day reliability of the laboratory results, conducting a seminar or providing training opportunities on laboratory internal quality control and on total quality management could be effective strategies to help in enhancing the knowledge and overall practice towards internal quality control amongst the laboratory personnel. Conducting a multicentric study involving laboratory personnel from different institutions would help us access the real knowledge status of laboratory personnel regarding laboratory IQC for better patient outcomes.

## CONCLUSIONS

The prevalence of knowledge of internal quality control for laboratory tests among laboratory personnel working in the department of biochemistry was similar to the other study done in a similar setting. Although the working experience is substantial, the laboratory personnel did not receive adequate training on laboratory internal quality control after the completion of their education. Hence, providing training opportunities on laboratory internal quality control can be reflected as a necessity in our current laboratory set-up. This could add value to the knowledge of IQC on laboratory personnel to ensure that the reports generated within the laboratory are accurate, reliable and reproducible.
